# *covR* Mediated Antibiofilm Activity of 3-Furancarboxaldehyde Increases the Virulence of Group A Streptococcus

**DOI:** 10.1371/journal.pone.0127210

**Published:** 2015-05-15

**Authors:** Ganapathy Ashwinkumar Subramenium, Dharmaprakash Viszwapriya, Prasanth Mani Iyer, Krishnaswamy Balamurugan, Shunmugiah Karutha Pandian

**Affiliations:** Department of Biotechnology, Alagappa University, Science Campus, Karaikudi, Tamil Nadu, India; University of Oklahoma Health Sciences Center, UNITED STATES

## Abstract

**Background:**

Group A streptococcus (*GAS*, *Streptococcus pyogenes*), a multi-virulent, exclusive human pathogen responsible for various invasive and non-invasive diseases possesses biofilm forming phenomenon as one of its pathogenic armaments. Recently, antibiofilm agents have gained prime importance, since inhibiting the biofilm formation is expected to reduce development of antibiotic resistance and increase their susceptibility to the host immune cells.

**Principal Findings:**

The current study demonstrates the antibiofilm activity of 3Furancarboxaldehyde (3FCA), a floral honey derived compound, against GAS biofilm, which was divulged using crystal violet assay, light microscopy, and confocal laser scanning microscopy. The report is extended to study its effect on various aspects of GAS (morphology, virulence, aggregation) at its minimal biofilm inhibitory concentration (132μg/ml). 3FCA was found to alter the growth pattern of GAS in solid and liquid medium and increased the rate of auto-aggregation. Electron microscopy unveiled the increase in extra polymeric substances around cell. Gene expression studies showed down-regulation of *covR* gene, which is speculated to be the prime target for the antibiofilm activity. Increased hyaluronic acid production and down regulation of *srtB *gene is attributed to the enhanced rate of auto-aggregation. The virulence genes (*srv*, *mga*, *lux*S *and hasA*) were also found to be over expressed, which was manifested with the increased susceptibility of the model organism *Caenorhabditis elegans* to 3FCA treated GAS. The toxicity of 3FCA was ruled out with no adverse effect on *C*. *elegans*.

**Significance:**

Though 3FCA possess antibiofilm activity against GAS, it was also found to increase the virulence of GAS. This study demonstrates that, *covR* mediated antibiofilm activity may increase the virulence of GAS. This also emphasizes the importance to analyse the acclimatization response and virulence of the pathogen in the presence of antibiofilm compounds prior to their clinical trials.

## Introduction

*Streptococcus pyogenes*, also called group A streptococcus (GAS) is a β-haemolytic pathogen which exclusively affects human and naturally inhabits human skin and throat [1,2]. It ranks among top ten infectious pathogens, affecting 700 million individuals and causing mortality over 500,000 per year globally [3]. It is an originator of wide number of invasive and non-invasive diseases like pharyngitis (strep throat), necrotizing fasciitis and streptococcal toxic shock syndrome [4]. An early treatment for streptococcal infection is imperative, since untreated mild infections can even lead to complex diseases like rheumatic heart disease and glomerulonephritis [5]. Virulence factors of GAS include hyaluronic acid capsule, pyrogenic exotoxins (A, B & C), surface associated M protein, streptokinase, streptodornase, streptolysin S, streptolysin O, and biofilm formation [6]. Biofilm formation is a protective effort put in by microbes to escape from antibiotics. In some cases, the biofilm-associated resistance to antibiotics is due to the inability of drug to penetrate the biofilm [7] and in other cases, antibiotics penetrate biofilms but are not effective compared to planktonic cells due to metabolic/physiological differences [8].

The extracellular matrix, three dimensional structure and difference in gene expression are the three barriers that prevent drug penetration and increases antibiotic resistance in microbes during biofilm mode of growth [9]. The emergence of erythromycin and clindamycin resistant GAS among healthy school children in Korea were found to be increased by the rate of 21.6% and 23.6% respectively, between 1995 and 2002 [2]. Erythromycin resistance was also observed with 44% in Finland during 1992 [10], 32% in USA during 1994–95 [11] and 17.1% in Spain during 1996 [12]. Tetracycline resistance strains (34%) were also observed among patients with invasive diseases in USA.

A number of studies have reported the biofilm formation of Group A Streptococus both *in vitro* and *in vivo* [13, 14, 9]. Biofilm formation of GAS has been linked to therapeutic treatment failure. For instance, all of the 99 GAS isolates obtained from the children between 2–18 years of age in Calgary, Canada, were found to possess biofilm forming ability and 32 of them did not respond to penicillin treatment [9]. About 90% of the total 289 clinical isolates of GAS causing both invasive and non invasive infections were found to possess biofilm forming efficacy [15]. Among the 60 children who were about to undergo tonsillectomy, 21 individuals (37%) were screened positive for GAS and SEM analysis of their tonsil revealed the presence of GAS to be in biofilm [16]. Thirty seven percent of non-severe recurrent acute otitis media cases in children were identified to be caused by nasopharyngeal biofilm- producing GAS [17].

The emergence of antibiotic resistance phenomenon among GAS and the clinical importance of streptococcal biofilm is an alarming threat to the mankind, which makes it mandatory to find novel antagonistic agents. Unlike antibiotics which build selective pressure on microbes and induce antibiotic resistance, these antagonistic agents are used to inhibit the pathogenicity of the organism than killing it. Antibiofilm compounds are one such class of compounds which inhibit the microbial biofilm formation, thereby aiding antibiotic penetration.

Two component systems (TCS) play a key role in adaptation of the microbes to varying environmental conditions [18]. GAS consists of about 13 such two way component systems among which covRS system is well studied and found to control about 15% of its total gene expressed [19,20]. GAS also consists of various stand-alone transcription factors which coordinate the virulence factor expression in the organism. *mga* and *srv* are such important stand-alone factors in GAS which play key role in its virulence factor expression [21]. Hence studying the influence of antibiofilm agents on these TCS and stand-alone regulators is expected to provide little insights on the target of the antibiofilm agents.

3-Furancarboxaldehyde (3FCA) is a volatile compound present in floral honey [22–24], fruits [25,26] and oils of certain plant roots [27]. 3FCA was also found to possess anti-oxidant property [28]. The current study aims to unveil the antibiofilm activity of 3-furancarboxaldehyde (3FCA) against GAS, its influence on virulence factor production and to investigate the mechanism governing its antibiofilm activity by studying the differential gene expression between control and 3FCA treated GAS. The *in vitro* studies were further manifested in *Caenorhabditis elegans*, a simple and widely used model organism for host-pathogen interaction and toxicological studies.

## Materials and Methods

### Ethical statement

In the current study, healthy human blood was used in blood survival assay and sheep blood was used in haemolysin quantification assay. Blood samples were used only for research purpose. The blood sample from healthy human (one of the authors of the manuscript) was drawn by technically skilled persons and a written consent was obtained. Experimental methodology and use of healthy human blood was assessed and approved by Institutional Ethical Committee (Human Research), Alagappa University, Karaikudi under the No. IEC/AU/2014/3. Fresh sheep blood sample was collected from the Karaikudi municipality modern slaughter house, Karaikudi. Since, the blood is normally discarded in the slaughter house; no specific permission from ethical board was required.

### Bacterial strain and culture condition

*S*. *pyogenes*, SF370 was cultured in tryptose agar (Hi-media, Mumbai, India) and todd hewitt broth (Hi-media, Mumbai, India) supplemented with 0.5% yeast extract and 0.5% glucose (THYG), and incubated at 37°C. Overnight culture of GAS with 0.4 OD at 600 nm was considered as standard cell suspension.

### 3-Furancarboxaldehyde

3-Furancarboxaldehyde (3FCA) (Catalogue no. W501603, Sigma Aldrich, USA) was prepared as 10 mg/ml stock solution dissolved in acetone and stored at 4°C till further use.

### Effect of 3FCA on GAS biofilm formation

3FCA was added at increasing concentrations individually to wells containing one ml of THYG, in a sterile 24 well micro titre plate. One per cent inoculum from standard cell suspension of SF370 was added to each well and incubated at 37°C for 24 h. After incubation, optical density (OD) of each well was measured at 600 nm, so as to check the effect of compounds on the growth of GAS SF370. Planktonic cells were then discarded and the wells were washed with sterile distilled water and allowed to dry. Dried wells were stained with 1ml of 0.4% crystal violet (w/v) for 10 minutes, washed twice with distilled water and allowed to dry. Biofilm bound crystal violet was then extracted using 20% glacial acetic acid for 10 min and the contents of the wells were read at 570 nm. The amount of dye present after washing directly reflects the amount of cells in the biofilm. Inhibition percentage was measured using the formula given below:

%ofinhibition=[(ControlOD570nm−TreatedOD570nm)/ControlOD570nm]*100

### Growth curve and viability of GAS

In order to assess the influence of 3FCA on growth of GAS, OD 600 of GAS cultures were observed over a period of 24 h at a regular interval of 1 h. The Viability of GAS was quantified using XTT-menadione. XTT-menadione solution was prepared freshly prior each experiment by adding 0.2 mg/ml XTT (Sigma Aldrich, USA) and 0.172 mg/ml menadione (Hi-media, Mumbai, India) at 12.5:1 ratio. Briefly, equal number of cells were inoculated to 1 ml of media in a 24 well microtiter plate with and without 3FCA and incubated at 37°C for 24 h. After incubation the planktonic cells were aspirated, harvested and washed with sterile PBS and resuspended in 200 μl of PBS. In order to collect cells involved in biofilm, each well was added with 200 μl of sterile PBS and scrapped thoroughly. To these planktonic and biofilm cell suspensions, 25μl of XTT-menadione suspension was added and incubated in dark at 37°C for 4 h. Cell viability is correlated to the reduction of XTT into orange colored formazan, which was quantified spectrophotometrically at 490 nm. The relative graph was drawn with planktonic and biofilm cell viability.

### Effect of 3FCA on GAS growth on solid and liquid media

Standard cell suspension of GAS was streaked over tryptose agar containing acetone (vehicle control) or 3FCA at MBIC concentration and incubated for 48 h at 37°C, in order to study its nature of growth on solid media. To investigate its growth in liquid media, 1% standard cell suspension was added to 1ml of THYG with and without 3FCA and incubated for 18 h at 37°C.

### Microscopic techniques

In order to study the architecture of biofilm upon treatment, biofilms were developed on 1 X 1 cm glass slides, which were placed in a 24 well plate containing 1ml of medium, 1% inoculum and compound at increasing concentrations. The plate was incubated for 24 h at 37°C. The slides were stained and fixed accordingly.

For light microscopic analysis, glass slides were washed with sterile PBS and stained with 0.4% crystal violet, which was then washed and air dried. The air dried slides were viewed under microscope (Nikon Eclipse 80i, USA) at 400X magnification and imaged.

For Confocal Laser Scanning Microscopy, the slides were washed with sterile PBS and stained with 0.1% acridine orange for 5 min at dark, and de-stained with sterile distilled water. The acridine orange stained cells were observed and imaged under CLSM (LSM 710, Carl Zeiss, Germany).

For scanning electron microscopy, the slides were washed with sterile PBS and fixed with 2% glutaraldehyde for 8 h at 4°C and washed with sterile PBS. The slides were then dehydrated with ethanol in increasing concentrations (20, 40, 60, 80 and 100%) and gold coated prior their observation under SEM (VEGA 3 TESCAN, Czech Republic).

For transmission electron microscopy, the 24 h liquid cultures of GAS grown in the presence and absence of 3FCA were centrifuged and the cells were washed with sterile PBS and fixed with 2% glutaraldehyde for 6 h at 4°C. Glutaraldehyde fixed cells were washed thrice with distilled water and a drop of the sample was placed on a piece of parafilm in a carbon coated copper grid of 3 mm diameter and allowed to dry. The grid was washed with distilled water and excess of water was removed. The dried grid was stained with 2% uranyl acetate, air dried and observed under TEM (Hitachi, H-7500, Japan).

### Auto-aggregation assay

Bacterial aggregation was calculated by visually observing the time it takes to settle at the bottom. Before the assay, GAS cultures were grown to 0.4 OD at 600 nm in the presence and absence of 3FCA. The cells were then harvested and resuspended in sterile PBS. The tubes were kept undisturbed and were visually observed every 5 minutes in order to check the rate at which the cells settle. Faster the cells settle, higher is the aggregation rate.

### Protease quantification

Azocasein assay was performed with control and compound treated GAS supernatant in order to quantify the total cysteine protease production. GAS was grown in the presence and absence of 3FCA for 24 h at 37°C. The culture was centrifuged at 12000 rpm, and the supernatants were filter sterilised through 0.2 micron nylon membrane filter. Equal volume of supernatant and activation buffer (1 mM EDTA, 20 mM DTT in 0.1 M sodium acetate buffer, pH 5.0) was added to the cell free culture supernatant and kept at 40°C for 30 min. To the mixture, equal volume of 1% (w/v) azocasein was added and incubated further for 1h at 40°C. Trichloro-acetic acid (10%) was then added and mixed thoroughly in order to precipitate the protein and stop the reaction. The mixture was then centrifuged at 12000 rpm for 5 min and the supernatant was read at 366 nm.

### Hemolysin quantification

Freshly collected sheep blood was washed twice with sterile PBS and resuspended to a final concentration of 2% (v/v) in PBS. Equal volumes of bacterial cell free culture supernatant and 2% blood were added together and incubated at 37°C for 1h followed by incubation at 4°C for 1h. The tubes were then centrifuged at 3000 rpm for 5 min and the supernatant was read at 405 nm in order to quantify the haemolysis that has occurred.

### Hyaluronic acid quantification

In order to isolate cell associated hyaluronic acid, 1% of standard cell suspension was inoculated in 1 ml of THYG containing acetone or 3FCA and incubated at 37°C for 24 h. The cells were then harvested and washed with sterile PBS and resuspended in 1 ml of PBS. To extract hyaluronic acid from cells, 1 ml of the suspension was added with 1 ml of chloroform and vortexed thoroughly and kept undisturbed at room temperature for 1 h. The suspension was then centrifuged and supernatant was quantified for hyaluronic acid. Cell associated hyaluronic acid was quantified using stains-all reagent (Sigma Aldrich, USA), which was prepared as described earlier [29]. Briefly, 20 mg of stains-All reagent was dissolved in a solution containing 50 ml formamide, 50 ml distilled water and 16ml of acetic acid. To 100 μl of supernatant, 1 ml of freshly prepared stains-all reagent was added, vortexed and the absorbance was read at 640 nm.

### Blood survival assay

Equal volume of overnight culture of GAS grown in the presence and absence of 3FCA was added to healthy human blood at 1: 4 ratio and mixed gently by inverting the tube and incubated at 37°C for 3 h and the total number of viable GAS was quantified by spread plate method.

### Total RNA isolation and cDNA Synthesis

Total RNA of GAS was isolated from the mixture of planktonic and biofilm cells collected at mid log phase in the presence and absence of compound at MBIC using Trizol reagent. The first-strand cDNA were synthesized using High Capacity cDNA Reverse Transcription Kit (Applied Biosystems, USA).

### Real Time PCR (qPCR) Analysis

RT-PCR was followed by real-time PCR (7500 Sequence Detection System, Applied Biosystems Inc. Foster, CA, USA) in a single-well format in which *S*. *pyogenes* gene-specific primers of *covS*, *covR*, *srv*, *mga*, *speB*, *lux*S, *hasA*, *ciaH*, *sclB*, *srtB*, *spy_125* and *gyrA* genes were combined separately with the PCR mix (SYBR Green kit, Applied Biosystems, USA) at a predefined ratio. Housekeeping gene, gyrase (*gyrA*) was taken as internal control. The role of these genes and their primer sequences are given in S1 Table. The PCR cycle number was titrated according to the manufacturer’s protocol to ensure that the reaction was well within the linear range of amplifications. The steady-state levels of candidate genes were assessed from the Ct (cycle threshold) values of the candidate gene relative to the Ct values of *gyrA* (internal control).

### *C*. *elegans* survival assay

*C*. *elegans* survival assay was done to unveil the toxicity of the compound if any, as well as to analyse the impact of 3FCA on the virulence level of GAS against *C*. *elegans*. Countable numbers (~10) of hermaphrodites at L4 stage were taken for the study. In order to assess the toxicity of the compound, *C*. *elegans* were grown in 1 ml of liquid medium (M9 buffer) containing *E*. *coli* OP50 (~ 1000 CFU/ml) (laboratory food source) in the presence and absence of 3FCA at MBIC. *C*. *elegans* grown with *E*. *coli* OP50 along with acetone (vehicle) was used as control.

In order to assess the influence of 3FCA on virulence of GAS, the survival rate of *C*. *elegans* grown in the presence of GAS + 3FCA was compared with that grown in presence of GAS + acetone (vehicle control). The inoculum load of GAS was ~1000 CFU/ml and 3FCA was used at MBIC (132 μg/ml). *C*. *elegans* were checked for every 4 h to assess their viability. The animals were considered to be dead when it showed no more response or movement to external stimuli like a gentle tap or touch with platinum loop.

### CFU assay

In order to assess the internalization of GAS in *C*. *elegans*, a total of 10 worms at L4 stage were grown for 24 h in tubes containing 1 ml of M9 buffer,132 μg of 3FCA and inoculated with 1% standard cell suspension of GAS. Worms grown in the presence of acetone (vehicle) and GAS was considered as control. The exposed *C*. *elegans* were washed first with M9 buffer containing 0.01% sodium azide. This was followed by a wash with tetracycline (0.5 μg/ml) in order to remove the skin adhered GAS. The washed live worms were crushed with silicon carbide (220 mesh) for the extraction of internalized GAS. The extracted cells were serially diluted and spread plated on tryptose agar plate. The plates were incubated overnight at 37°C, and the total CFU were counted.

### Statistics

All the experiments were performed at least twice in independent experiments in triplicates. All data were expressed as arithmetic mean ± standard deviation. Unpaired Student's t-test was used to compare the groups. Statistical significance was set at P<0.01.

## Results

### Antibiofilm and non-fatal effect of 3FCA on GAS

**Fig 1:**
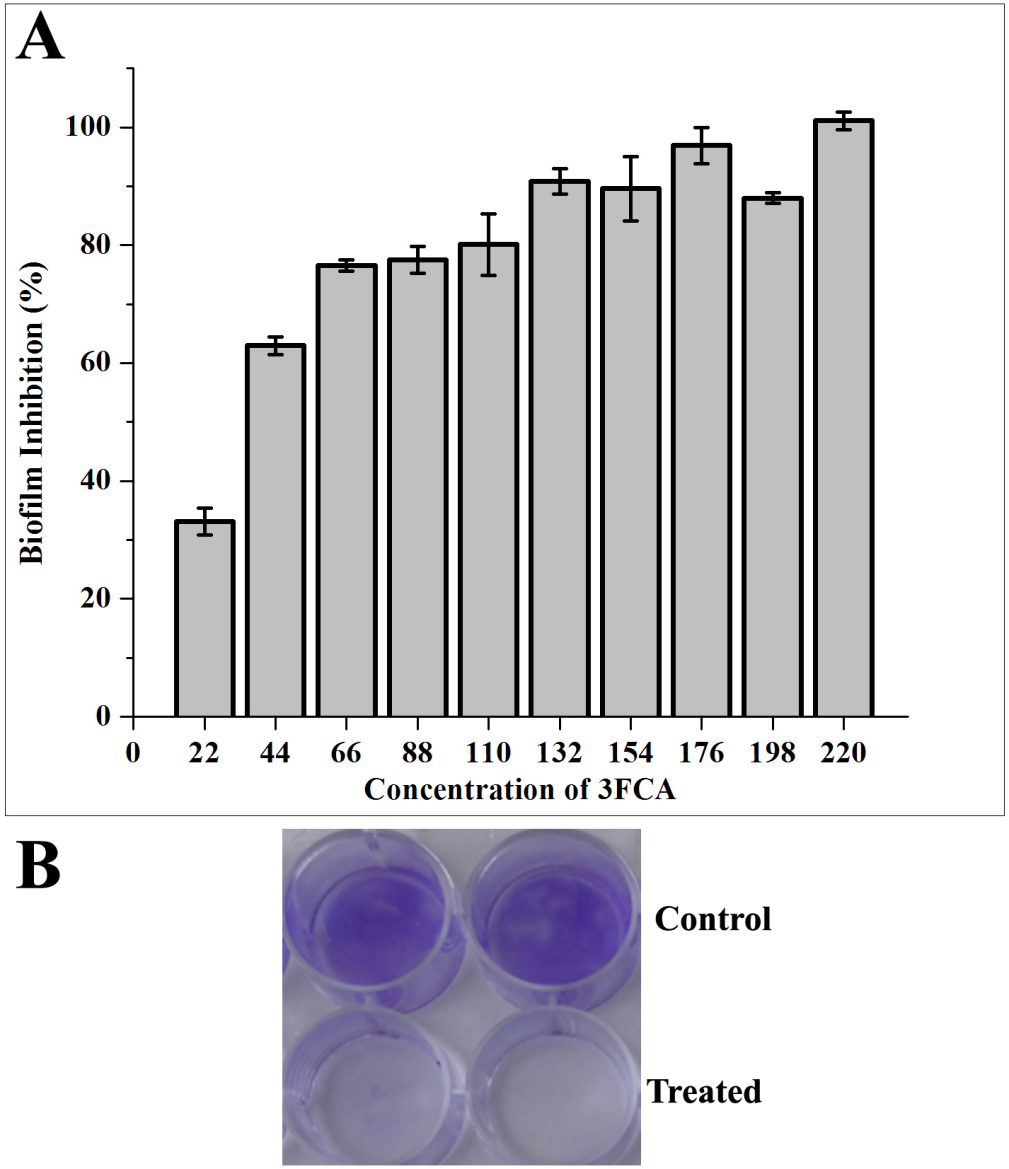
Quantification of biofilm formation by crystal violet staining. Biofilms were grown in 24 well micro titre plates for 24 h in 1 ml THYG, with increasing concentrations of 3FCA or acetone (vehicle control). **(A)** Percentage inhibition of GAS biofilm grown in presence of 3FCA in comparison with that grown in presence of vehicle control (acetone). Significant increase in biofilm inhibition was observed with increasing concentrations of 3FCA. Error bars indicate standard deviations. The presence of 3FCA at 132 μg/ml was found to reduce 90% of group A streptococcal biofilm formation. **(B)** Photograph showing biofilms grown with (bottom panel) and without (top panel) 3FCA at MBIC (132μg/ml) after crystal violet staining.

**Fig 2:**
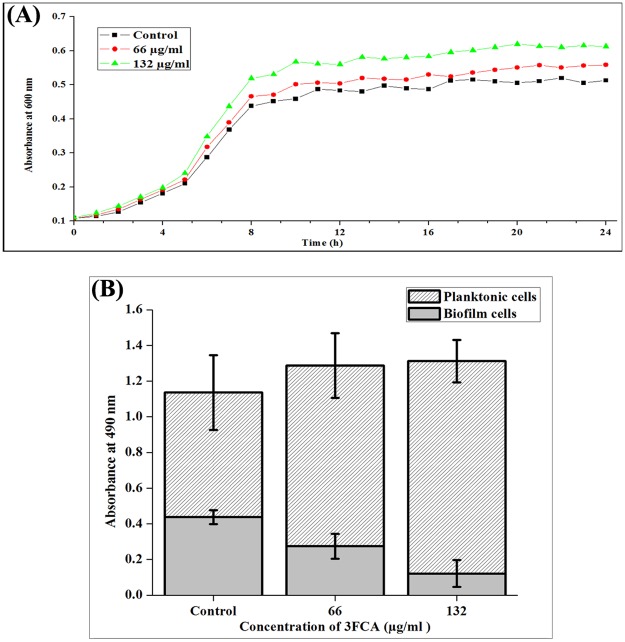
Growth of GAS in the presence and absence of 3FCA at MBIC. **(A)** A representative graph depicting the OD 600 of cultures of GAS grown in the presence and absence of 3FCA at MBIC and 1/2MBIC. The doubling time of GAS in the presence and absence of 3FCA were found to be around 2 h and the cells reached stationary phase at the eleventh hour. **(B)** The viability of control and 3FCA (132 μg/ml) treated biofilm and planktonic GAS were quantified after 24 h using XTT reduction assay. An insignificant difference was found in the viability of GAS in control and treated samples.

The antibiofilm activity of 3FCA was measured in a microtiter plate using crystal violet. A concentration dependent increase in antibiofilm activity was observed. 3FCA at 132 μg/ml concentration showed 90% activity. Since no significant increase in the activity was observed at higher concentrations, 132 μg/ml was considered as Minimum Biofilm Inhibitory Concentration (MBIC) (Fig 1). The growth curve of GAS in the presence and absence of 3FCA also showed a mild increase in the OD 600 nm of 3FCA treated samples compared to control and the doubling time of GAS in both the control and 3FCA treated cultures were found to be around 2 h and the cells reached the stationary phase at eleventh hour itself. The non antibacterial activity of 3FCA was also assessed by quantifying and comparing the total number of viable cells in the control and treated wells using XTT. The results showed no significant difference among the total viable cells in the control and treated samples, which confirmed that 3FCA does not have any antibacterial activity against GAS (Fig 2). The total number of viable cells involved in biofilm and planktonic mode of growth, were quantified individually. The total number of cells involved in biofilm formation of 3FCA treated samples was reduced, wherein the absorbance was found to be 0.12 and that in control was found to be 0.4. Complementing this, the viable planktonic cells were found to be increased from 0.7 OD in control to 1.25 OD upon treatment. On comparing the absorbance at 490 nm of biofilm cells in the control and treated samples, it was found that only 26% of cells were allowed to form biofilm in treated samples.

### Microscopic techniques

**Fig 3:**
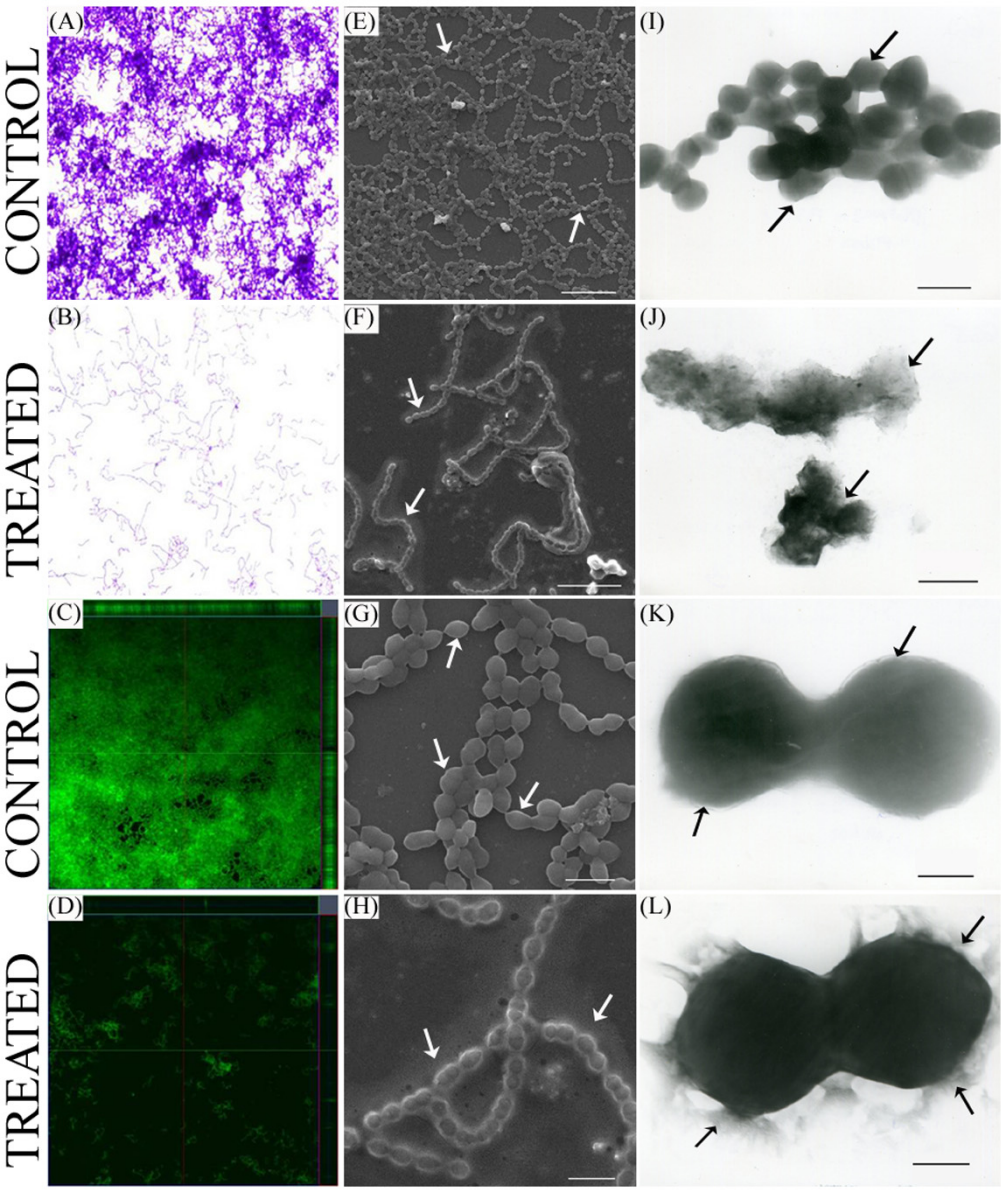
Microscopic analysis of GAS. Microscopic images of GAS grown over glass slides (1 x 1 cm) in THYG in the presence and absence of 3FCA at 132μg/ml concentration. **(A, B)** Light microscopic images (at 400X) of crystal violet stained GAS biofilm grown in the presence of 3FCA (B) and in presence of vehicle control (A). **(C, D)** CLSM images of acridine orange stained group A streptococcal biofilm grown in the presence (D) and absence (C) of 3FCA. Both the light microscopic and CLSM images clearly display the antibiofilm activity of 3FCA. **(E, F, G, H)** SEM images of GAS biofilm. (E and F) showing the breach in streptococcal biofilm grown in the presence of 3FCA. Scale-10 μm. (G and H) Displaying the difference in the morphology of GAS grown in the presence (H) and absence of 3FCA (G). Scale-2 μm. (**I, J, K, L)** TEM images displaying the increased extracellular hyaluronic acid secretion around 3FCA treated GAS (J, L) compared to corresponding control (I, K). [I, J Scale-1 μm, K, L scale-250 nm]. Significant differences in control and treated samples are highlighted with arrows.

The light microscopic as well as CLSM images clearly showcase reduction in the biofilm covered surface area on treatment with 3FCA. The biofilms were also studied under SEM, which showed decreased chains or biofilm on treatment. The morphology of the organism was also observed to be modulated. The treated cells were observed to be coated with increased extracellular matrix compared to control. The framework of the cell wall of control and treated cells was found to be dissimilar. TEM analysis was performed in order to avoid dehydration of the cells and to visualize them in their native form. The images clearly displayed increased hyaluronic acid secretion around 3FCA treated cells (Fig 3).

### Effect of 3FCA on morphology of GAS

**Fig 4:**
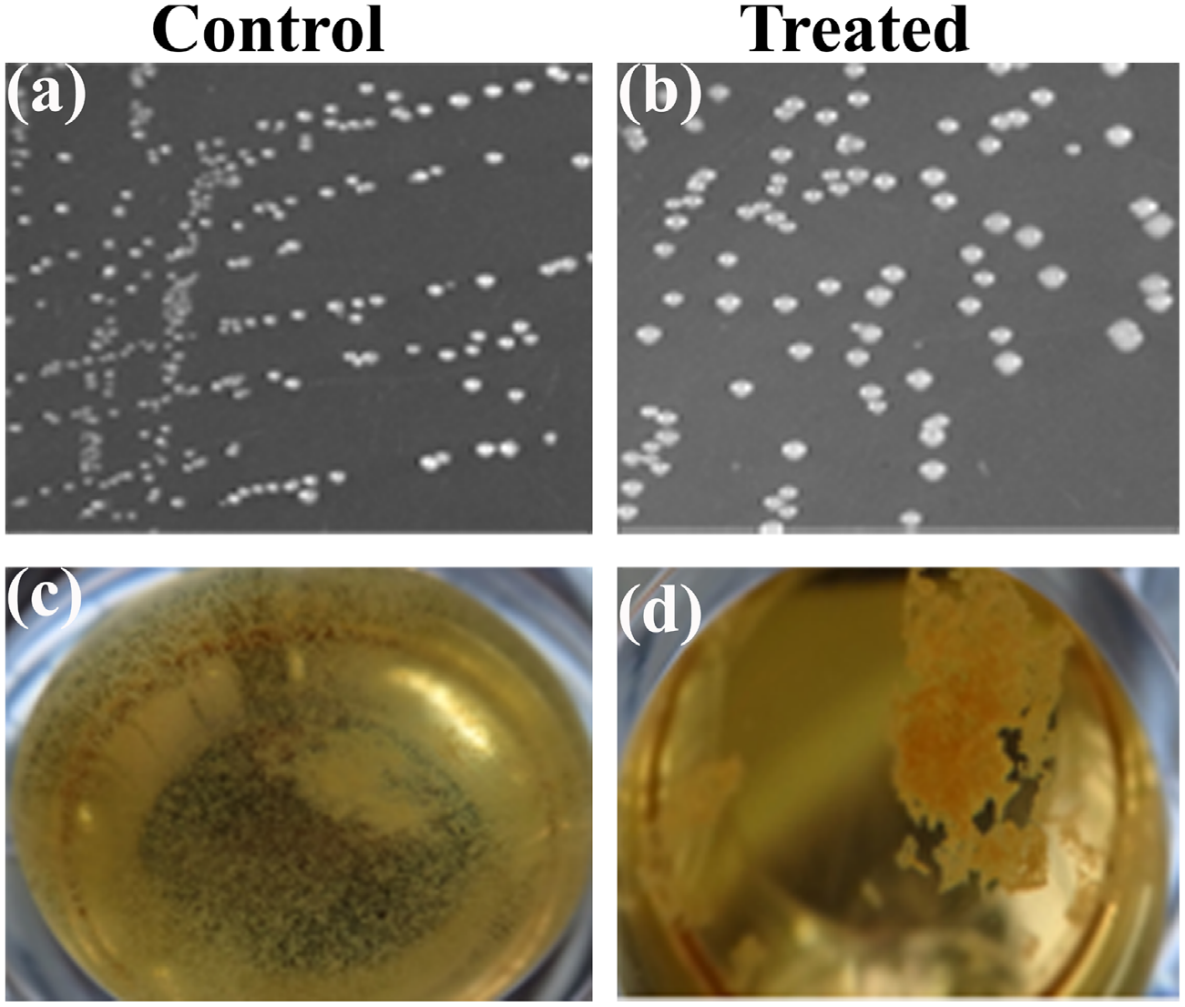
Morphology of GAS grown in tryptose agar. **(a, b)** Growth of GAS in Tryptose agar plates. GAS growth pattern were found to be mucoid in the presence of 3FCA (b) compared to its control (a). **(c, d)** Growth of GAS in liquid medium (THYG). GAS grown in the presence of 3FCA (d) were found to be clumped at the centre, whereas their corresponding control (c) were uniformly distributed throughout the well and found to be adhered to the surface.

The colonies of 3FCA treated GAS on agar plates were found to be more mucoid and bigger in size, compared to its control and those grown in liquid media were seen to be in unusual clumps and non adherent to the surface which is in total contrast to the control cells settled at the bottom, with uniform growth pattern throughout the well (Fig 4). The change in the morphology was also confirmed with TEM analysis which showed that treated cells were covered with hyaluronic acid.

### Effect of 3FCA on auto-aggregation pattern

**Fig 5:**
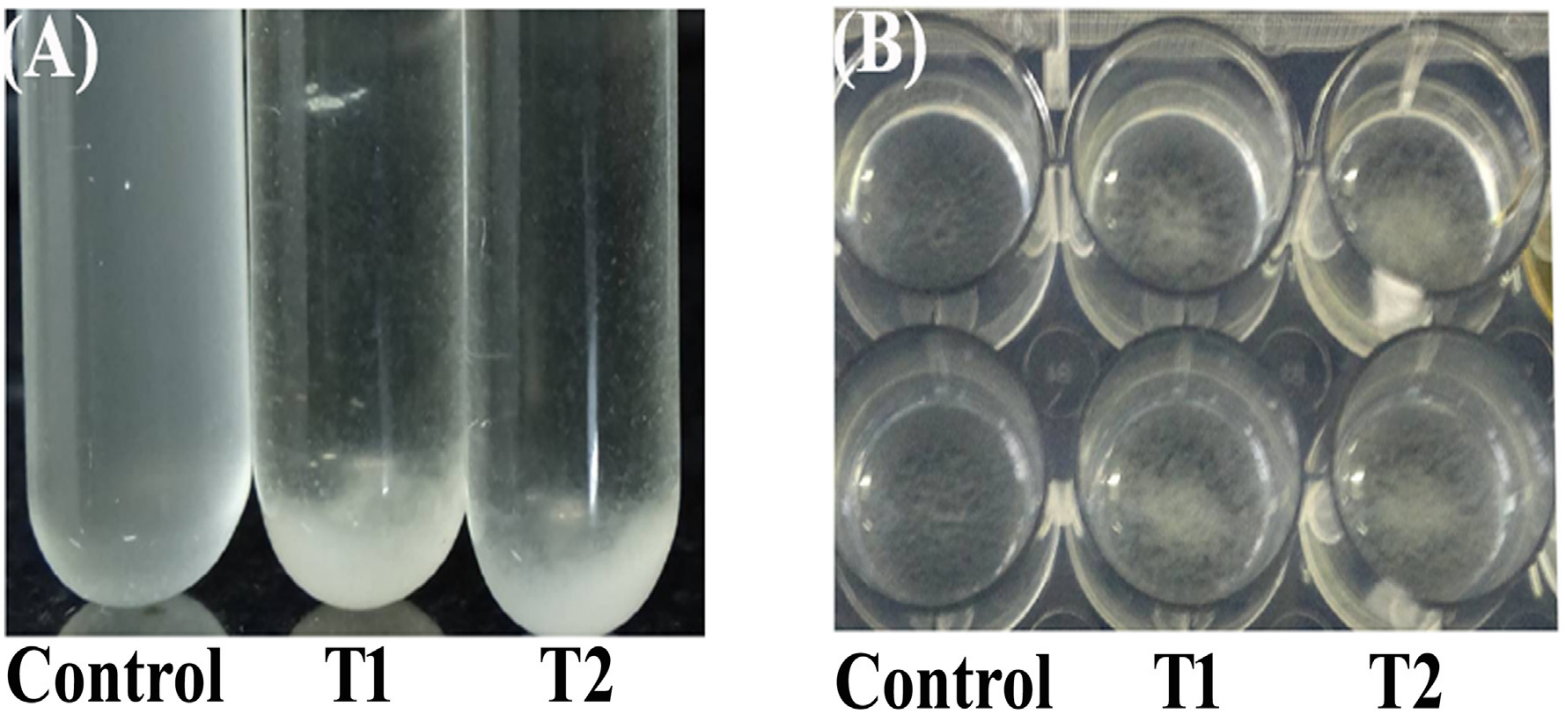
Auto-aggregation pattern of GAS. Photograph showing auto-aggregation patterns of GAS suspensions in the absence of 3FCA (Control), presence of 3FCA at 1/2 MBIC (66 μg/ml-T1) and MBIC (132 μg/ml-T2) after 30 min in test tube (A) and in micro titre plate (B). An increased rate of auto-aggregation was observed among treated samples in both the cases.

Auto aggregation assay results showed drastic aggregation of 3FCA treated GAS. The level of bacterial aggregation was examined by measuring the time taken for the organism to settle. During the time course, the turbidity of untreated control had no difference till 30 min, whereas 3FCA treated cells were observed with increased rate of aggregation. Even after one hour of incubation in static state, control cells did not settle whereas, treated cells were found to settle within 30 min. Similar result was also observed in polystyrene plate. Aggregation of 3FCA treated cells was observed at the center of the plate whereas no difference was seen in untreated wells. Images were obtained after incubating the plate and tubes for 30 min in static condition (Fig 5).

### Effect of 3FCA on protease and haemolysin production

Extra cellular cysteine protease is an important and well-studied virulence factor of GAS. On the other hand, streptolysin O and streptolysin S are the oxygen labile and oxygen stable exotoxins responsible for the hemolytic activity of GAS. The results indicated no significant difference in protease and haemolysin production upon treatment with 3FCA indicating that 3FCA was found to be ineffective against extracellular protease and haemolysin production by GAS (Table 1).

**Table 1:** Effect of 3FCA on the production of extracellular virulence factors (protease, heamolysin) and survival of GAS in healthy human blood.

**Proteolysin Quantification**	**Absorbance at 366 nm (Mean ± SD)**			
	Control	T1		T2
	0.532 ± 0.027	0.539 ± 0.009		0.535 ± 0.006
**Heamolysin Quantification**	**Absorbance at 405 nm (Mean ± SD)**			
	Control	T1		T2
	1.431 ± 0.004	1.363 ± 0.018		1.291 ± 0.006
**Survival of cells in healthy human blood**	**Total number of cells/ml ± SD**			
	Control		Treated	
	2.4 ± 0.1 x 10^7^		2.3 ± 0.2 x 10^7^	

Control—Vehicle control (acetone), T1- 1/2MBIC (66 μg/ml 3FCA), T2- MBIC (132 μg/ml 3FCA).

### Hyaluronic acid quantification

**Fig 6:**
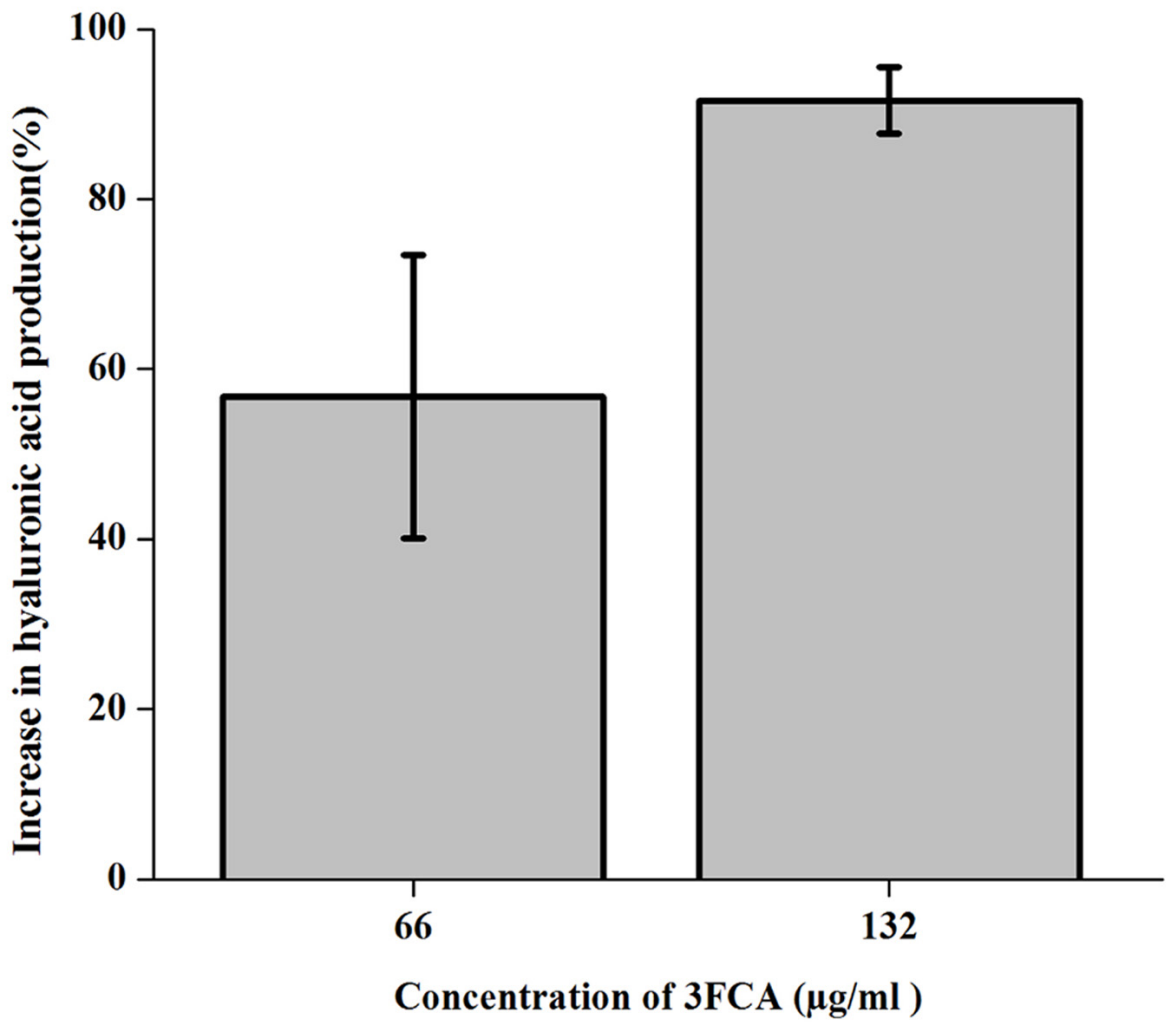
Hyaluronic acid quantification of GAS. Percentage increase in hyaluronic acid production by GAS grown in the presence of compound with respect to control. Error bars indicate standard deviations. A concentration dependent increase in hyaluronic acid production was observed.

Hyaluronic acid capsule which act as a shield for GAS in escaping from the human immune system was quantified using Stains all reagent. The results showed a concentration dependent increase in the hyaluronic acid secretion of up to 91% on treatment with 132μg/ml of 3FCA (Fig 6).

### Effect of 3FCA on *ex vivo* blood survival

Hyaluronic acid capsule and surface associated M protein, which are responsible for the escaping of the organism from phagocytosis was primitively assessed with its survival in healthy human blood. The results showed an insignificant difference among the survival of control and treated GAS in healthy human blood (Table 1).

### Real time PCR (qPCR) analysis

**Fig 7:**
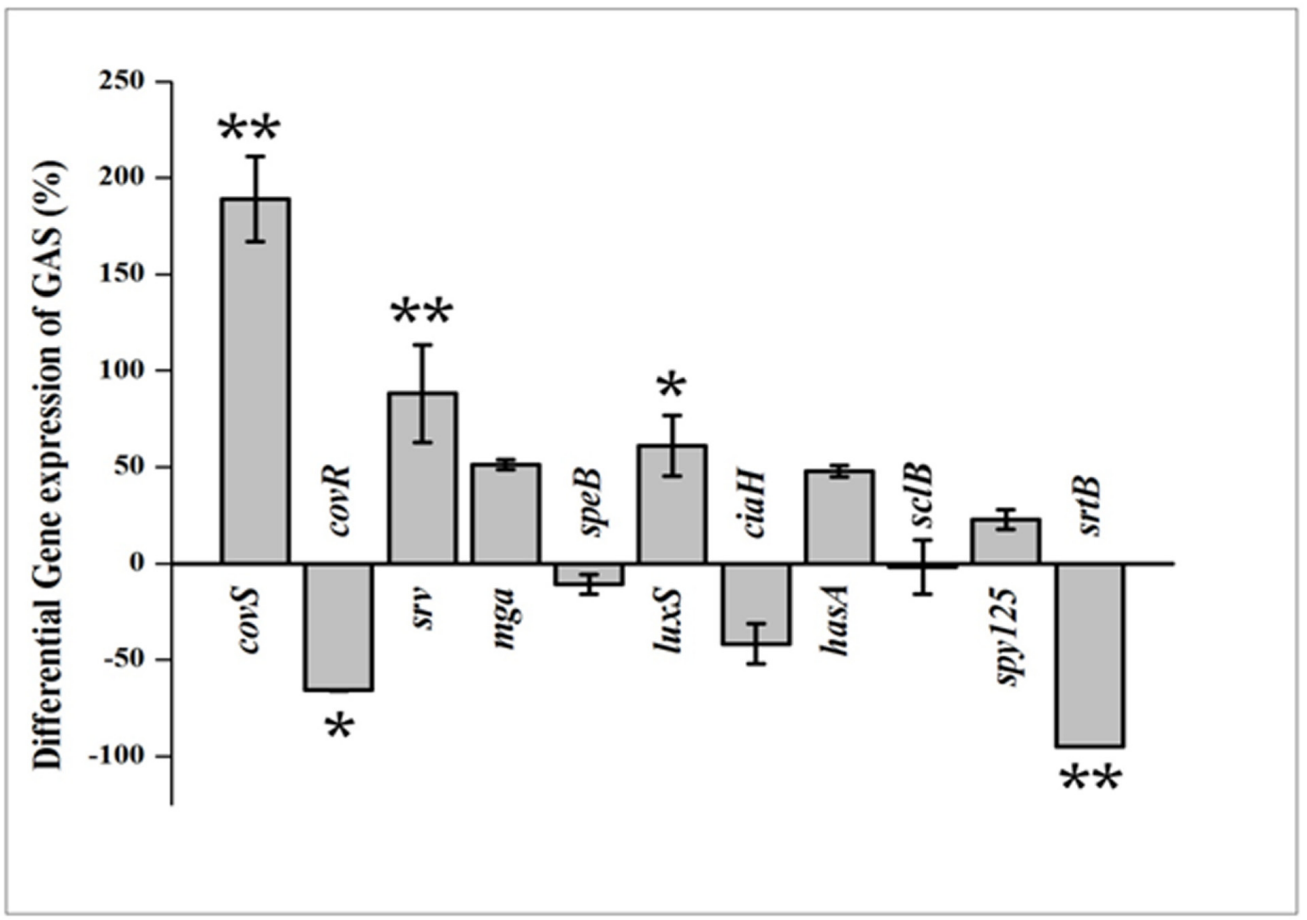
Differential Gene expression pattern. Effect of 132 μg/ml of 3FCA on relative gene expression of specific genes involved in virulence, biofilm formation and aggregation in GAS. Data are expressed as mean ± standard deviations. The expression was normalized to the house keeping gene *gyrA*. * indicates p <0.005 and ** p<0.001 compared to control.

To find out the effect of 3FCA on the gene expression pattern, qPCR was performed. The expression of genes involved in TCS (*covRS*, *ciaH*), stand-alone regulators of virulence (*mga*, *srv*), quorum sensing (*lux*S) and streptococcal exotoxin B production (*speB*), hyaluronic acid synthesis (*hasA*), cell wall associated protein synthesis (*srtB/spy_135*), streptococcal collagen like protein synthesis (*sclB*) and minor pilin subunit synthesis (*spy_125*) were studied. All these candidate genes were known to be directly or indirectly involved in streptococcal virulence, biofilm formation and aggregation. Detailed descriptions of the genes, their role and the nucleotide sequences of primers used in the study are given in the supplementary S1 Table. The gene expression levels of the treated cells were compared with control and normalised to one. The results displayed significant down regulation in the expression of *covR*, *srtB* and *cia*H (66%, 95% and 41% respectively) and up regulation in the expression of *cov*S, *lux*S, *mga*, *srv and hasA* (189%, 61%, 51%, 88% and 48% respectively) genes. An insignificant difference in the expression levels was observed for *speB* and *sclB* genes (Fig 7).

### *C*.*elegans* survival assay

**Fig 8:**
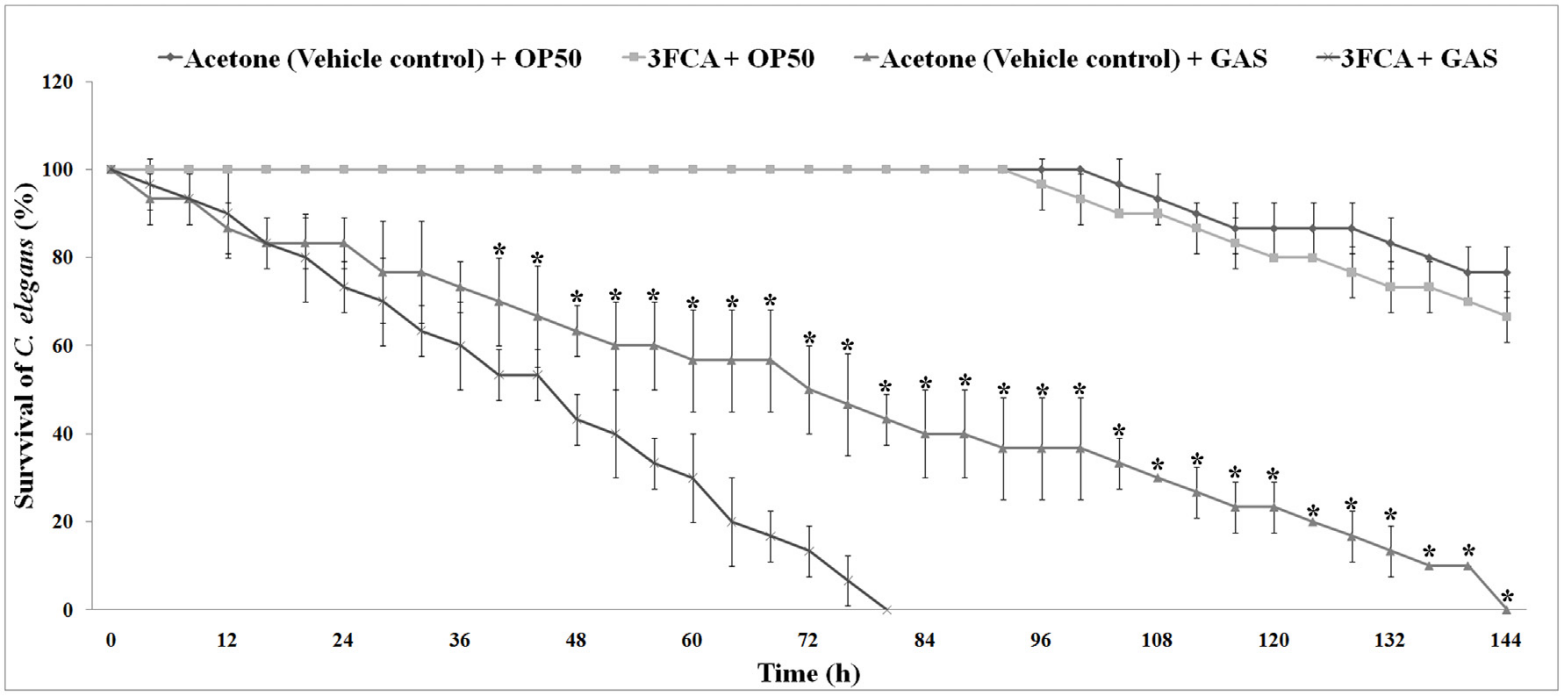
Effect of 3FCA and 3FCA treated GAS on the survival of *C*. *elegans*. *C*. *elegans* were exposed to Acetone (vehicle control) + OP50 (♦), 3FCA + OP50 (■), Acetone (vehicle control) + GAS (▲), 3FCA + GAS (**×**). The survival rate of C. elegans in presence of OP50 was found to be insignificantly affected by 3FCA, whereas significant differences were observed among the lifespan of the nematodes grown in the presence of GAS alone when compared to those grown in 3FCA treated GAS. * denotes p<0.001 compared to control.

A compound should be nontoxic in nature so as to be used at clinical level. To confirm this, cytotoxicity of 3FCA was assessed on a simple, eukaryotic model organism, *C*. *elegans*, which is widely used in compound screening and toxicological studies. The avirulent *E*. *coli* OP50, uracil auxotroph was used as food source for the model. Acetone + OP50 and 3FCA + OP50 were used respectively as control and test sample. The results revealed that 3FCA had no cytotoxic effect on *C*. *elegans*. In order to assess the streptococcal virulence level, Acetone + SF370 and 3FCA + SF370 were used as control and treated samples respectively. In presence of GAS, complete killing took place in 144 h, whereas the nontoxic 3FCA in combination with GAS showed much reduced survival time of only 80 h for complete killing of *C*. *elegans* (Fig 8).

### CFU assay

In order to confirm the enhanced virulence of 3FCA treated GAS, enumeration of GAS internalised by *C*. *elegans* in its intestine was performed. As expected, the total number of GAS CFU obtained from the intestine of *C*. *elegans* incubated with 3FCA treated GAS were found to be 2.47 x 10^5^ CFU/ml compared to 4.23 x 10^4^ CFU/ml in untreated GAS, which is approximately six fold increase in internalisation.

## Discussion

In the current era, emergence of multi drug resistance phenomenon among microbes has made a dire need for novel antagonistic agents which are not dreadful to microbes but reduces the human risk factors associated with pathogenicity. One such class of modern microbial antagonistic agents are called antibiofilm compounds, which inhibit the microbial biofilm formation. Biofilm formation is considered as one of the most important virulent factors of microorganism which help them to survive in hostile conditions and prevents penetration of antibiotics to the cells encased in it [30]. Unlike antibiotics, these antibiofilm compounds do not exert selection pressure on microorganisms; thereby antibiotic resistance phenomenon can be eliminated. Nevertheless, as the organism survives in the environment where its biofilm is depleted, it becomes important to investigate its behaviour and pathogenecity in the presence of the antibiofilm agents and unveil their mode of action. The present study investigates the antibiofilm activity of 3-Furancarboxaldehyde, a volatile compound present in floral honey against Group A Streptococcus and a great deal of attention has been paid to study the effect of 3FCA on biofilm formation and virulence factor production. The current study demonstrates that, 3FCA possesses strong antibiofilm activity against GAS. It is hypothesised from our results that 3FCA targets the *covRS* TCS leading to down regulation of *covR* which in turn increases the virulence of the organism. 3FCA also promotes aggregation of the organism which is speculated to be due to the combinatorial effect of increase in hyaluronic acid production and down regulation of *srtB* gene. The hypothesis is supported with both physiological assays and gene expression studies

The antibiofilm efficacy of 3FCA was evaluated against GAS, which showed a concentration dependent increase in the activity. The effect of 3FCA was also studied *against S*. *mutans*, *S*. *mitis*, *S*. *salivaris* and *S*. *sanguinis* which revealed that 3FCA neither pose antibacterial nor antibiofilm activity against any of these pathogens (Data not shown). The obtained results clearly suggest that 3FCA possesses specific antibiofilm activity against GAS. Monitoring the growth curve of the organism for 24 h showed a dose dependent increase in the absorbance, which when verified with XTT assay showed an insignificant difference. The results revealed 3FCA as an ideal antibiofilm agent against GAS with no antibacterial effect. Light microscopic analysis showed reduction in biofilm covered surface area in the 3FCA treated wells and CLSM analysis revealed decrease in the thickness of biofilm.

The SEM micrograph of treated cells showed an abnormal morphology, which prompted us to study the morphology of GAS grown in solid media in the presence of 3FCA and its behaviour in liquid media. The result of SEM analysis was corrugated by the mucoidal CFUs seen in tryptose agar plates supplemented with 3FCA. Even in liquid media, treated cells were found to be grown in clumps and floating contrary to control samples. The difference in the cell surface was also analysed with TEM which clearly showed mucous layer surrounding the cells. The layers surrounding the cells in SEM analysis is attributed to be dehydrated mucous secreted by the cells. Earlier studies reveal that the mucoidal nature of the organism is associated with the production of M protein and hyaluronic acid capsule [31,32]. This lead us to quantify the cell associated hyaluronic acid and M protein. Cell wall associated hyaluronic acid was quantified with Stains-All reagent which showed increased hyaluronic acid production as the concentration of 3FCA increases. In order to further corroborate the results, GAS was treated with healthy human blood, since hyaluronic acid capsule and surface associated M protein play a crucial role in evading opsano-phagocytosis [33]. The results showed that the treated cells were also equally able to grow in healthy human blood, which suggest that the mucoidal nature of 3FCA treated GAS is due to increased production of cell wall associated M protein and hyaluronic acid capsule. This result goes in parallel with the real time PCR data wherein *mga* and *hasA* were found to be over expressed. *mga* is a gene which regulates *emm* gene production and *hasA* is a gene involved in hyaluronic acid capsule synthesis [34].

On the other hand, aggregated nature of growth of 3FCA treated GAS in liquid culture triggered us to study the auto-aggregation pattern of GAS in the presence and absence of 3FCA. 3FCA treated GAS showed an increased rate of auto-aggregation than untreated cells. The GAS cells which are encapsulated with higher amount of hyaluronic acid were found to grow in clumps and were seen to settle down easily, since they are oxygen sensitive [35]. A similar phenomenon was seen here where 3FCA treated cells were found to grow in clumps and aggregate rapidly. Hence, increase in auto-aggregation is ascribed to the increased hyaluronic acid production. This increase in aggregation is also expected to be the cause of antibiofilm activity since an increased binding efficacy was seen between the organisms than binding to the substratum. The effect of 3FCA on the protease and hemolysin production of GAS was also explored and found to be insignificant. A previous study reports about 50% antibiofilm activity and increased aggregation pattern of *S*. *pyogenes* MGAS6180 on treatment with morin hydrate at 225 μM concentration [36].

In order to unveil the mode of antibiofilm activity of 3FCA against GAS and to test whether the antibiofilm activity of 3FCA is also manifested at transcriptional level, the expression level of candidate genes involved in biofilm formation, aggregation, virulence factors and surface associated proteins were quantified. Two component regulatory systems (TCS) and stand-alone factors are considered to be the prime regulators for the expression of the streptococcal armament of virulence genes. Among the genes used in the study, *covR* and *covS* genes are the repressor and sensor kinase genes respectively [37] involved in *covRS* TCS which is well-studied and characterised in GAS [1]. Cov is the acronym of Cluster Of Virulence in streptococci [38] which actively or passively influences about 15 per cent of overall genes encoded by GAS [39]. *covR* was initially considered as the major negative regulator of genes involved in hyaluronic acid capsule production (*hasA*, *hasB & hasC*) [40] and it rapidly responds to the changes in the environment [41]. The results showed down regulation of *covR* gene, and up regulation of *covS* and *hasA* gene which goes in parallel with the earlier report, in which *covR* mutant SF370 was observed to have eight fold higher expression of *covS* compared to its corresponding wild type [42]. The study also demonstrates over expression of *hasA* by *covR* mutant strain. The up regulation of *hasA* in the expression studies was also reflected in the physiological assay, which together confirms the increased hyaluronic acid production. The inability of 3FCA treated GAS to form biofilm and down regulation of *covR* gene goes in parallel with the previous report in which *covR* mutants were found to lack biofilm forming ability [43].

*covR* is a repressor gene which represses various virulence genes like *mga* (Multiple Gene regulator of GAS), *spe*B, *hasA* and *luxS* under normal condition [44–46]. Hence, it becomes clear that down regulation of *cov*R has led to the up regulation of *mga* and *lux*S. LuxS is an enzyme of activated methyl cycle which in GAS influences virulence gene production in a growth dependent manner [47]. The up regulation of *mga* which is a positive regulator of M gene protein expression [48] and global transcriptional activator in exponential growth phase was confirmed with the growth of treated GAS in healthy human blood. The results of survival in healthy human blood is also supported by a previous report wherein no significant difference in *in vivo* growth rate between wild type and *cov*R mutant strain in rat was observed [49]. *srv* is another important gene called streptococcal regulator of virulence which plays an active role in streptococcal virulence and knock out mutation of which inhibits the biofilm forming efficacy of GAS [50]. To our surprise, *srv gene* on treatment was found to be increased, which means the compound may increase the virulence of GAS.

*speB* is a gene encoding extracellular protease production, which on over expression may act on its biofilm by lysing the proteins involved in biofilm [50]. There was no significant difference in the expression pattern of *speB*. Nevertheless, an earlier report states that *covR* mutation increases *speB* production [42], but no such increase was reflected in protease quantification as well as in *speB* expression. *srv* and *covR* exert opposite influence on *speB*, in which the former inhibits and the latter induces *speB* production [50,42]. From the finding of the current study, it is hypothesised that, up regulation of *srv* would nullify the impact of down regulation of *covR* on *speB* gene expression thereby, causing no significant change in extracellular protease production.

*ciaH*, an another important TCS which controls about 120 genes [21] and involved in oxidative stress and acid tolerance [51] was found to be down regulated. An earlier report which demonstrated the increased aggregation pattern in *srtB* mutant M6 strain [52] aroused great interest in us to study the gene expression pattern of *spy_125/srtB* gene in SF370 which was found to be down regulated. Down regulation of *srtB* gene is also attributed to the increased rate of cell aggregation.

On the note that 3FCA being unexplored for its medicinal application, the toxicity of 3FCA was assessed primitively, with the help of eukaryotic model organism *C*. *elegans*, which is widely used for ecotoxicology studies as a living bio monitor [53]. *C*. *elegans* also conserves many of the basic physiological processes and stress responses observed in humans [54]. The unaffected growth of *C*. *elegans* in the presence and absence of the compound confirms that 3FCA does not have any lethal effect on the growth of *C*. *elegans* and hence can be considered as a nontoxic compound. The gene expression studies which showed 3FCA treated GAS to be more virulent than the untreated ones, was also confirmed with the help of the same model organism. The rate in which *C*. *elegans* were killed by treated GAS was much higher to that of untreated control which strengthens the gene expression data of increased virulence production of GAS in the presence of 3FCA. The increased hyaluronic acid production and increase in adherence of treated GAS were expected to help their internalisation in the *C*. *elegans*, which was confirmed with the CFU assay.

In summary, we demonstrated the antibiofilm activity of 3FCA, a compound from floral honey, and its major influence in the morphology and virulence of GAS. Our study suggests that 3FCA may target the *covR* gene of well-studied covRS pathway, which is a negative regulatory pathway controlling virulence genes of GAS. The down regulation of *covR* is hypothesised to be the prime reason for the observed biofilm inhibition since most of the studied genes (*covS*, *hasA*, *and mga*) were found to express in a similar pattern like that of *covR* mutant SF370 which lacks biofilm forming ability [43]. The increase in virulence was confirmed using physiological assays, gene expression analysis and *in vivo* studies in *C*. *elegans*. The present study is in disagreement with the outlook that antibiofilm agents which inhibit the biofilm would also reduce the virulence of the organism. Nevertheless, the *in vivo* increase in the virulence of the organism upon 3FCA treatment needs to be further investigated in higher eukaryotic model organism. In addition, the present study emphasizes the importance of analysing the behaviour and virulence of the pathogens in presence of the antibiofilm compounds ahead of clinical studies.

## Supporting Information

S1 Table List of genes, their role and the nucleotide sequences of primers used in the study. This file contains the list of genes, their role and the nucleotide sequences of primers used in gene expression analysis.
(DOCX)
