# Supplementary material for: covR Mediated Antibiofilm Activity of 3-Furancarboxaldehyde Increases the Virulence of Group A Streptococcus
Source: PLoS One. 2015 May 15;10(5):e0127210. doi: 10.1371/journal.pone.0127210 (PMC4433207; doi:10.1371/journal.pone.0127210)
Supplement: S1 Table — This file contains the list of genes, their role and the nucleotide sequences of primers used in gene expression analysis. (DOCX) [file pone.0127210.s001.docx]

**Table S1: List of genes, their role and the nucleotide sequences of primers used in the study**

| **Gene** | **Role** | **Primer Sequence (current study)** | |
| --- | --- | --- | --- |
|  |  | **Forward** | **Reverse** |
| *covS* | Stress response, biofilm formation and regulate 15% of streptococcal genome [1] | GAGTGAGCGCGATATCACAA | GCAAGCCAGGAGATGATTCT |
| *covR* |  | TGCGCGTGATTCTATTATGG | GGCGGAAAATAGCACGAATA |
| *mga* | Biofilm formation and virulence factor regulation [2] | GATCCGTTACTACAAGGG | GTTACTTGTCTGCCTCCT |
| *srv* | Biofilm formation and virulence factor regulation [3] | CGGCATTGTGAAACAGAGTG | TCTGACTCGATGCGAACATT |
| *luxS* | Virulence factor regulation [4] | CTTTTGGCTGTCGAACAGGT | TCCAGGAACATCTTCCCAAG |
| *speB* | Extracellular cysteine protease production [3] | CTAGGATACTCTACCAGCG | CAGTAGCAACACATCCTG |
| *hasA* | Hyaluronic acid capsule production [5] | AGCGTGCTGCTCAATCATTA | AACATCGATCATCCCCAATG |
| *ciaH* | Stress response TCS [6] | GGCGGTCTTACAGAATCGTC | CATGTTGCGAACCTCGTCTA |
| *sclB* | Collagen like surface protein production involved in aggregation [7] | GGTGACAAAGGCGATGCTGG | TGGCGCCGTCTTTACCTGGG |
| *srtB* | Class C sortase production involved in aggregation [8] | GCTGGTTTTGGTTTGTGGGA | CCCCGGGATATTTAACCAACC |
| *spy_125* | Minor pilin subunits on cell surface [9] | AGAGATTAGCGACGCAACAG | ATGGCCATATGTCTCCACCA |
| *gyrA* | Gyrase production (House-keeping gene) | CAACGCACGTAAGGAAGAAA | CGCTTGTCAAAACGACGTTA |

1. Graham MR, Smoot LM, Migliaccio CAL, Virtaneva K, Sturdevant DE, Porcella SF, Federale MJ, Adams GJ, Scott JR, Musser JM (2002) Virulence control in group A Streptococcus by a two-component gene regulatory system: global expression profiling and *in vivo* infection modeling. P Natl Acad Sci 99: 13855-13860.

2. Cho KH, Caparon MG (2005) Patterns of virulence gene expression differ between biofilm and tissue communities of *Streptococcus pyogenes*. Mol Microbiol 57: 1545-1556.

3. Doern CD, Roberts AL, Hong W, Nelson J, Lukomski S, Swords WE, et al. (2009) Biofilm formation by group A Streptococcus: a role for the streptococcal regulator of virulence (Srv) and streptococcal cysteine protease (SpeB). Microbiol 155: 46-52.

4. Lyon WR, Madden JC, Levin JC, Stein J, Caparon MG (2001) Mutation of *luxS* affects growth and virulence factor expression in *Streptococcus pyogenes*. Mol Microbiol 42: 145-157.

5. Levin JC, Wessels MR (1998) Identification of csrR/csrS, a genetic locus that regulates hyaluronic acid capsule synthesis in group A Streptococcus. Mol Microbiol 30: 209-219.

6. Tatsuno I, Isaka M, Okada R, Zhang Y, Hasegawa T (2014) Relevance of the two-component sensor protein CiaH to acid and oxidative stress responses in *Streptococcus pyogenes*. *BMC Research Notes* 7: 189-198.

7. Rasmussen M, Bjorck L (2001) Unique regulation of SclB–a novel collagen‐like surface protein of *Streptococcus pyogenes*. Mol Microbiol 40: 1427-1438.

8. Kimura KR, Nakata M, Sumitomo T, Kreikemeyer B, Podbielski A, Terao Y, et al. (2012) Involvement of T6 pili in biofilm formation by serotype M6 *Streptococcus pyogenes*. J Bacteriol 194: 804-812.

9. Manetti AG, Zingaretti C, Falugi F, Capo S, Bombaci M, Bagnoli F et al. (2007) *Streptococcus pyogenes* pili promote pharyngeal cell adhesion and biofilm formation. Mol Microbiol 64: 968-983.
